# In-vitro validated methods for encoding digital data in deoxyribonucleic acid (DNA)

**DOI:** 10.1186/s12859-023-05264-6

**Published:** 2023-04-21

**Authors:** Golam Md Mortuza, Jorge Guerrero, Shoshanna Llewellyn, Michael D. Tobiason, George D. Dickinson, William L. Hughes, Reza Zadegan, Tim Andersen

**Affiliations:** 1 Department of Computer Science, Boise State University, Boise, Idaho USA; 2 School of Materials Science, Boise State University, Boise, Idaho USA; 3 Department of Nanoengineering, Joint School of Nanoscience and Nanoengineering, North Carolina A&T State University, Greensboro, NC USA; 4 School of Engineering, Kelowna, University of British Columbia, Kelowna, British Columbia Canada

**Keywords:** Nucleic acid memory, DNA, Data storage, Information encoding, Error correction

## Abstract

Deoxyribonucleic acid (DNA) is emerging as an alternative archival memory technology. Recent advancements in DNA synthesis and sequencing have both increased the capacity and decreased the cost of storing information in de novo synthesized DNA pools. In this survey, we review methods for translating digital data to and/or from DNA molecules. An emphasis is placed on methods which have been validated by storing and retrieving real-world data via in-vitro experiments.

## Introduction

Based on an ever-increasing rate of data creation, the amount of global digital data is projected to reach 175 zettabytes (ZBs) by 2025 [1]. Currently, a data center capable of storing 1 exabyte (EB) requires hundreds of megawatts of power and more than 100 billion USD to build and maintain for 10 years [2]. Extrapolating these costs to 175 ZB projects costs of millions of megawatts and over 175 trillion USD just for information storage, which is not sustainable. Thus cheaper and more energy efficient technologies for storing digital information are needed to avert an information storage crisis [3].

With current technologies for electronic and magnetic memory approaching their physical and economic limits, it is unlikely that current approaches for reducing cost and increasing efficiency will successfully address these issues [1, 4]. However, living organisms exhibit highly efficient information storage systems, and methods using deoxyribonucleic acid (DNA) based information storage may prove efficient enough to address this crisis [4, 5]. These considerations are based on three key properties of DNA: (1) Under proper conditions, DNA is known to retain data for hundreds of thousands of years [6]. (2) An individual molecule of single-stranded DNA is theoretically capable of storing information at a density of 455 EBs per gram [7], implying that 0.5 kg of DNA could be sufficient to store all global digital data expected in 2025. (3) DNA’s energy of operation is many orders of magnitude less than current electronic memories (Table 1) [6]. Consequently, DNA based information storage has become an international research focus [2, 8, 9], incurred investment nationally [10, 11], and has been identified as an emerging industrial opportunity [6, 12].

**Table 1:** Comparison between established memory technologies and cellular DNA

Memory (*type*)	Retention (*years*)	ON power (*W*/*GB*)	Areal density ($bit/cm^{2}$)	Volumetric density ($bit/cm^{3}$)	Latency ($\mu s/bit$)	Error rate (error/bit)
Flash	10	0.01 – 0.04	$10^{10}$	$10^{16}$	100	$10^{-15}$ [7]
Hard drive	> 10	0.04	$10^{11}$	$10^{13}$	$3*10^3$– $5*10^3$	$10^{-15}$ [15]
Magnetic tape	30 [15]	0.004 [16]	$10^9$– $10^{10}$[19]	N/A	60–200 [19]	$10^{-18}$ – $10^{-21}$ [15]
Cellular DNA	> 100	$< 10^{-10}$	$10^{22}$	$10^{22}$[4]	$< 100$	$10^{-9}$ – $10^{-8}$ [20]

This table was adapted from the work of Zhirnov et al. [6]

## Survey of in-vitro validated storage methods

In his 1959 lecture titled “Plenty of Room at the Bottom”, Richard Feynman recognized the potential of using DNA to store information [13]. To the knowledge of the authors, information was first stored using synthetic DNA circa 1988 [14]. In the following decades, advances in DNA sequencing and synthesis technologies –primarily driven by the Human Genome Project– led to an increased interest in storing information in DNA. Numerous methods for writing and reading information from DNA have been reported since. In the following paragraphs, several recently reported representative methods with explicit in-vitro validation are introduced. The methods are presented in chronological order, showing how the field has progressed in terms of volume and complexity of data, and sophistication of storage methods. The encoding process used for in-vitro validation is restated as a procedure with the mapping algorithms (i.e., algorithms which convert from a sequence of digital values to a sequence of DNA bases) separated from other logic as much as possible.

**Fig. 1:**
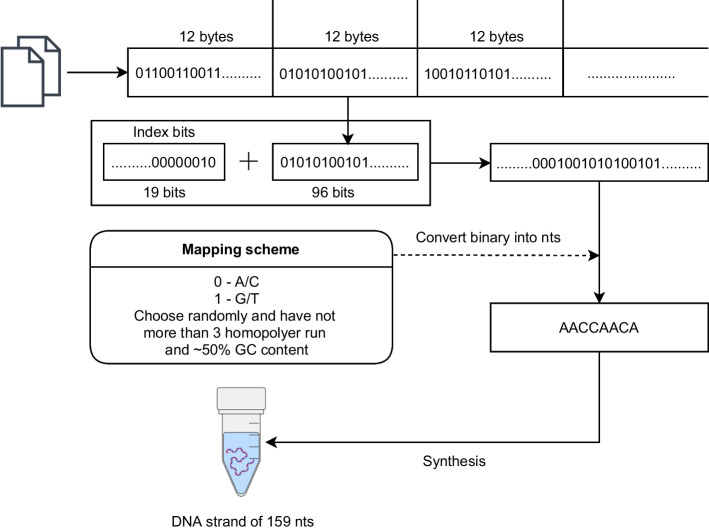
Diagram of the encoding algorithm reported by Church et al. [7]. Data is split into data blocks and attached to index bits. The resulting binary sequence is then directly mapped to a base sequence

The earliest of the methods was reported by Church et al. [7]. This method was used to store 659 kilobytes of data containing a book. The encoding process for this method can be described as the following procedure shown in Fig. 1. First, the data was split into sequentially addressed data blocks each containing 96 bits. Each block was then prepended with a 19 bit indexing address. This 115-bit sequence was coverted to a 115-base sequence by mapping 0 to Adenine (A) or Cytosine (C) and 1 to Guanine (G) or Thymine (T). The 115-base sequence was then flanked with a pair of 22 base primer sequences for amplification and sequencing purposes. This resulted in a set of 159-base sequences which collectively encode the data.

**Fig. 2:**
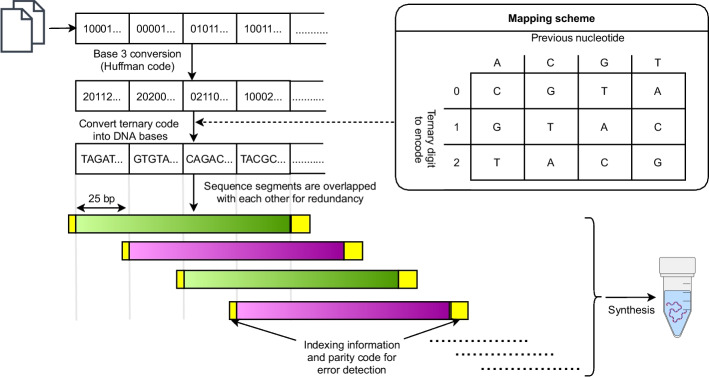
Diagram of the encoding algorithm reported by Goldman et al. [15]. A rotating mapping algorithm is used to avoid homopolymers. A parity code ensures the integrity of each segment. Every alternating segment is reverse complemented for data security (shown in violet color)

The next method was reported by Goldman et al. [15]. This method was used to store five files which totaled 757,051 bytes and included two text files, a pdf, a photograph, and an mp3. The encoding process for this method can be described by the following procedure shown in Fig. 2. The data was provided as a list of files. An index was assigned to each file and each file was represented as a sequence of bits (base-2 values). The bit-sequence for each file was converted to a sequence of trits (base-3 values) using a Huffman-code. This trit sequence was then converted to a base sequence according to a rotating mapping code (“Mapping Scheme” in Fig. 2). This yielded a single base sequence encoding the entire file, which was then split into indexed segments containing 100 bases and overlapping by 75 bases. The base sequence of every other segment was replaced with its reverse-complement (i.e., A/T swapped, G/C swapped, and then reversed). The following additional bases were then added to each 100-base sequence: (1) Two bases to indicate the file index, (2) twelve bases containing the index of the segment, (3) two bases indicating if the sequence has been reverse-complemented, (4) one parity base for detecting errors. This resulted in a set of 153,335 117-base sequences collectively encoding the data.

**Fig. 3:**
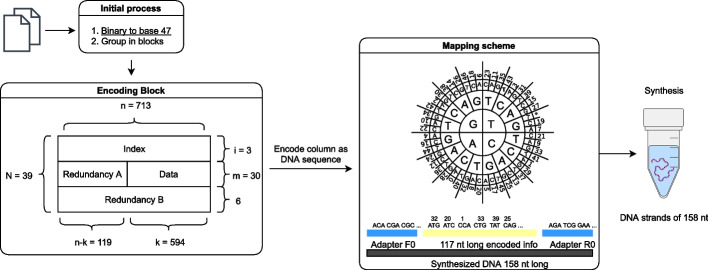
Diagram of the encoding algorithm reported by Grass et al. [16]. Binary data is converted to base 47 and packaged into $713 \times 39$ character matrices. Each column is then encoded as a DNA sequence with each character encoded as a codon according to the mapping scheme

The third method was reported by Grass et al. [16]. This method was used to store two text files totaling 83 kilobytes. The encoding process for this method can be described by the following procedure shown in Fig. 3. First, the digital data was converted into a number within the Galois Field (GF) of size 47 (GF(47)). These numbers were then put into a block of $594\times 30$ values (this can be seen in the encoding block of Fig. 3). The first RS parity information was added to each row in the form of 119 values from the GF(47). This section was referred to as the outer block (Redundancy A in the diagram). Next, an index section containing three values was added. Next, a second level of RS parity was added. This section containing six values was referred to as the inner block (Redundancy B in the diagram). Each column consisted of 39 base-47 values which were converted to a base sequence according to a word-based mapping code depicted by the wheel in Fig. 3. Two constant base-sequences used as primers were then attached to each 117-base sequence, yielding a set of 4,991 158-base sequences which collectively encode the data.

**Fig. 4:**
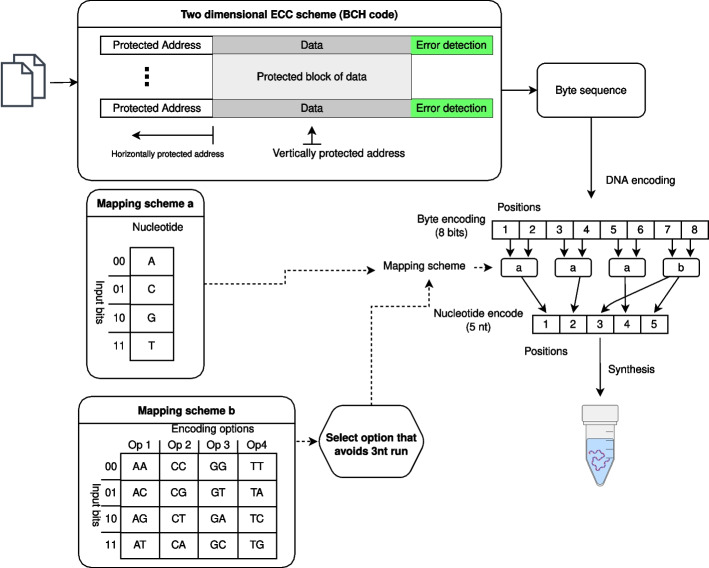
Diagram of the encoding algorithm reported by Blawat et al. [17]. The binary sequence is split into bytes, which are encoded to 5-base sequences using a combination of the two mapping schemes (a,b)

The fourth methods was reported by Blawat et al. [17]. This method was used to store a 22 megabyte video file. According to the understanding of the authors, the encoding process for this method can be described by the following procedure shown in Fig. 4. First, the digital file was split into segments of non-overlapping bit sequences. A 39-bit sequence used for segment addresses was encoded using a (63,39) Bose-Chaudhuri-Hocquenghem (BCH) code and prepended to the segment’s bit sequence. A 16 bit cyclic redundancy check code was then calculated and appended to the bit-sequence. The bit-sequence was then represented as a byte-sequence and converted to a base-sequence using the following mapping algorithm. The pre-determined mapping code associates each byte value with at least 2 and at most 3 5-base sequences. The first six bits of the byte determine the bases in positions 1, 2, and 4 depending on the table labeled “mapping scheme a” in Fig. 4. The last two bits of each byte encode bases 3 and 5 using the table labeled “mapping scheme b” in Fig. 4. Valid 5-base sequences satisfied two rules: 1) The first three bases cannot be the same, and 2) the last two bases cannot be the same. This mapping algorithm yielded 190-base sequences, to which a pair of 20 base primer sequences were attached. This resulted in 225,000 230-base sequences which collectively stored the data. The authors mention the presence of a Reed Solomon (RS) code to protect blocks of consecutive sequences, however, we were unable to determine the exact nature and location of this code from the text of the manuscript.

**Fig. 5:**
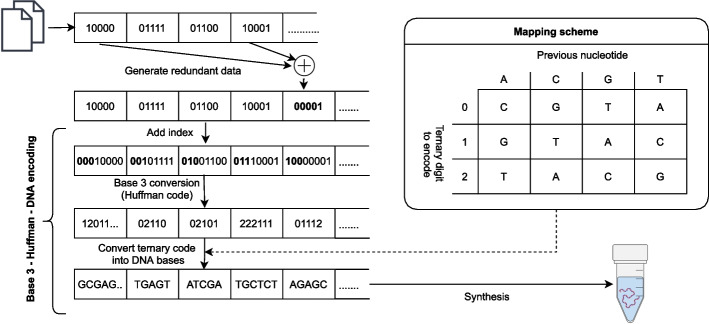
Diagram of the encoding algorithm reported by Bornholt et al. [19]. A key component of this method is the inclusion of the XOR operation. A rotating mapping algorithm was used to avoid homopolymers

The fifth method was reported by Bornholt et al. [18, 19]. This method was used to store four image files totaling 151 kilobytes of data. The encoding process of this method can be described by the following procedure shown in Fig. 5. First, a pair of base-sequences for use as primers and a base-sequence for use as a file address were chosen from an existing library. The bit sequence of the file was converted to a trit sequence using a Huffman code. The trit sequence was then converted to a base-sequence according to a rotating mapping code. This base-sequence was then split into non-overlapping segments. The following were then added to each segments: 1) the two 9-base primer sequences, 2) the file address sequence, 3) two bases to indicate if the sequence has been reverse-complimented. These sequences were then added to the list of sequences to synthesize. For redundancy, additional sequences were calculated by using an exclusive or operation to combine two segments into a single sequence. These additional sequences were included such that all segments were present in one direct sequence and one exclusive-or sequence.

**Fig. 6:**
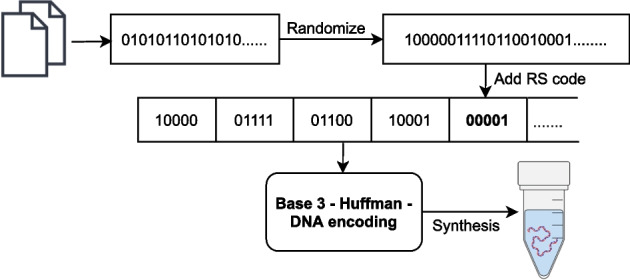
Diagram of the encoding algorithm reported by Organick et al. [20]. A key component of this method is the RS code used for generating redundant sequences

The sixth method was reported by Organick et al. [20]. This method was used to store 35 files totaling 200 megabytes of data. The encoding process for this method can be described by the following procedure shown in Fig. 6. First, the digital data was partitioned into files and each file was assigned a pair of 20-base sequences for use as primers. The bit-sequence of each file was then randomized by performing an exclusive or operation with bits generated from a pseudo random number generator. The randomized bit-sequence for each file was then partitioned into indexed rectangular matrices containing 16-bit cells. Each matrix contained 10 rows and up to 55,000 columns. For error correction, a RS code was applied to each row, and these bits were included as additional columns. Next, each column was treated as a sequence of bits and the address information (the matrix index and column index) were appended to this bit-sequence. This bit-sequence was converted to a trit-sequence, which was subsequently converted to a base-sequence using a rotating mapping code. Two 20-base primer sequences indicating the file index were then attached to each sequence.

**Fig. 7:**
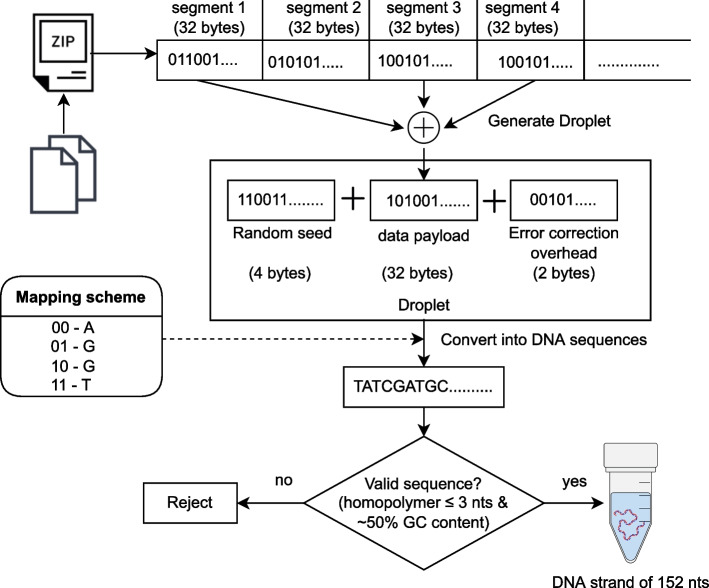
Diagram of the encoding algorithm reported by Erlich and Zielinski [21]. A combination of fountain code and RS code was used to provide robustness against dropout

The seventh method was reported by Erlich and Zielinski [21]. This method was used to store a single tarball file representing 2.14 megabytes of data. The encoding process for this method can be described by the following procedure shown in Fig. 7. First, the file was represented as a bit-sequence and partitioned into equally-sized, non-overlapping segments of 256-bits. A random 32-bit value was generated and used to initialize two Pseudo Random Number Generators (PRNG). The first PRNG was created over a Robust Soliton probability distribution and was used to choose the number of data segments to store in the droplet. The second PRNG was created over a uniform distribution and was used to select which segments to include in the droplet. The selected segments were combined into a single 256-bit sequence using an exclusive-or operation. The 32-bit seed was then prepended to the bit-sequence and the resulting 288-bit sequence was encoded using a RS code which appended 16 additional bits for error-correction. This 304-bit sequence was then converted to a 152-base sequence according to a direct mapping code where {00, 01, 10, and 11} map to {A, C, G, and T}, respectively. At this point, the base-sequence was rejected if it had unacceptable GC content or a long stretch of consecutive identical bases. Otherwise, the base-sequence was accepted and added to the list of valid base-sequences. Base-sequences were generated by repeating this process (starting at the generation of a new 32-bit seed) until 7% redundancy had been achieved. At this point, the 72,000 152-base-sequences encoded the 2.14 megabyte file at an information density of 1.57 bits/base. Two 24-base primer sequences were then attached to each base-sequence, bringing the length of each sequence to 200 bases and the information density down to 1.19 bits/base.

**Fig. 8:**
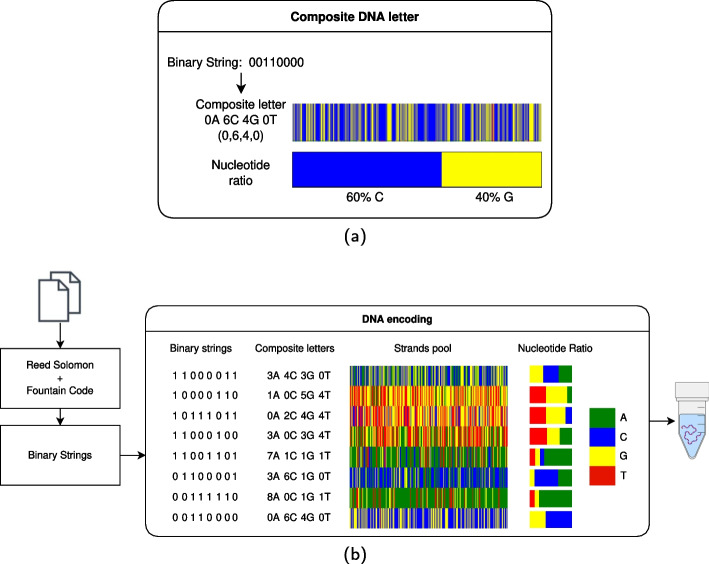
Diagram of the encoding scheme reported by Anavy et al. [22] **a.** Each 8-bit binary string is encoded as a DNA sequence containing a specific fraction of bases (60% C and 40% G in the example). **b.** A binary file is encoded using RS error correction and a Fountain Code. The droplets are then split into binary strings, which are converted into composite letters

An eigth method was reported by Anavy et al. [22]. This method was used to store a single zip file totaling 6.4 megabytes of data. The encoding process for this method can be described by the following procedure shown in Fig. 8. The following procedure specifically describes encoding into the 6-letter composite base alphabet. First, the file was represented as a bit-sequence and partitioned into equally-sized, non-overlapping segments of 320-bits. A random 28-bit value was generated and used to initialize two Pseudo Random Number Generators (PRNG). The first PRNG was a Robust Soliton distribution used to choose the number of data segments to store in a given droplet. The second PRNG was a uniform distribution used to select which segments to include in a given droplet. The selected segments were combined using the exclusive-or operation into a given droplet as a single 320-bit sequence. This 320-bit sequence was mapped to a sequence of composite bases according to a word-based mapping code which associated each 5-bit sequence with a 2-composite-base sequence. Composite bases do not represent a specific base (i.e., A, T, C, or G), but instead represent the distribution of bases at this position following synthesis. The formal definition of composite bases can be found in the work of Anavy et al. [22]. A single base was appended to this 128-composite-base sequence to bring it’s length to 129 bases. A systematic RS code over Galois field $7^3$ was used to append 6 composite-bases of error correction to this sequence, bringing its total length to 135 bases. A RS code over Galois field $4^2$ was used to append 4 bits of error correction to the 28-bit random seed, bringing this bit-sequence to 32-bits. This 32-bit sequence was then converted to a (non-composite) 16-base sequence according to a direct mapping code where 00, 01, 10, and 11 map to A, C, G, and T, respectively. The 16-base sequence was appended to the 135-composite-base sequence, yielding a 151-base sequence containing both composite and non-composite bases. At this point any base-sequences were rejected which contained composite letters not in the original 6-letter composite alphabet. The following base-sequences were then attached to the 151-base sequence: 1) a pair of 20 base sequences used as primers, and 2) a single 3-base sequence used to identify the file or experiment. Multiple droplets (with associated random seed, error correction code, primers, and file ID) were generated by repeating this process (starting at the generation of a new 28-bit seed) until 8% redundancy had been achieved.

More recently, Ping et al [23] report a method, the Ying Yang Coding algorithm (YYC), that eliminates long homopolymer runs (allowing for runs of at most 3 nt). Their algorithm also rejects encoded strands with gc content less than 40% or greater than 60%, and strands with free energy greater than − 30 kJ mol$^{-1}$. The algorithm achieves this through a flexible codec based on Goldman’s rotating code that provides a total of 1536 different transcoding schemes for encoding binary sequences.

The algorithm starts by segmenting the binary file into equally sized segments. This is followed by an interative loop where two segments are randomly selected at the beginning of the loop. For each pair of selected segments, each bit of the both segments are processed sequentially, with the first segment’s *i*th bit used to choose one of two possible nucleotide pairings, where 0 maps to 1 of two possible nt combinations, and 1 maps to the remaining two nt combinations (there are 6 possible nt pairings for this codec). This is followed by the application of a rotating code that selects a nt pairing based on the last encoded nt and the *i*th bit of the 2nd segment (giving 256 possible encodings at this step). The intersection of these two nt pairs is chosen as the next nt in the encoded output (the construction of the codecs ensures that there is only one nt in the intersection of these two nt pairs), and the process iterates. The process terminates and rejects the encoded sequence if the gc content of the encoded sequence falls outside of a prescribed range, if a homoplymer run of 4 or greater is detected, or if the free energy is greater than than − 30 kJ mol$^{-1}$. One drawback of their approach is that only 65% of randomly selected segment pairs are able to pass their screening tests in general, and this percentage drops drastically for extremely 0 or 1 biased file segments (although this can be mitigated by compressing the file before processing).

## Recurring or notable strategies

Information has been stored in DNA base-sequences using a variety of methods, including those surveyed in section "Survey of in-vitro validated storage methods". However, certain themes are present in most, if not all, of these methods. The following sections discuss several notable themes and the different strategies used to address them.

### Codes mapping a digital-sequence to a base-sequence

In one sense, all methods discussed here encode digital-data to base-sequences. However, for most methods, there exist a sub-process (i.e., a mapping algorithm) where a sequence of digital values is converted to a sequence of DNA bases such that a set of encoding rules (i.e., a mapping code) is satisfied. The following notable mapping codes were found in the literature.

Some known methods utilize mapping codes where a single digital value maps to a single base-value [21, 22, 24]. Referred to as direct mapping codes, such codes are notable primarily due to their simplicity and the fact they can sometimes be applied without a decrease in information density. For example, the method reported by Erlich and Zielinski [21] utilizes a mapping code where {0, 1, 2, 3} are mapped to {A, C, G, T}, respectively.

Some known methods utilize mapping codes where a digital value can map to one of several bases. The primary benefit of such mapping codes is the freedom to map a given digital-sequence to one of several base-sequences, which can be used to select experimentally desirable sequences (i.e., balance GC content, avoid secondary structures, or eliminate homopolymers). The primary disadvantage of such mapping codes is the decrease in information density they cause. One example of such mapping codes is present in the method reported by Church et al. [7]. In this specific code, 0 is mapped to A or C, and 1 is mapped to G or T. This results in a 50% reduction in information density.

Several known methods utilize mapping codes where a digital value maps to a specific base-sequence, or one of several base-sequences [16, 17, 22]. Referred to as word-based mapping codes, such codes can enable the exclusion of undesired base-sequences (such as homopolymers) at the cost of decreased information density. One example of a word-based mapping code is present in the method reported by Grass et al. [16]. The value/base-sequence mappings for this code are depicted using a wheel-like diagram and shown in Fig. 3.

Several known methods utilize mapping codes where a digital value is mapped to a base depending on context (such as the value of the previous base)[15, 18–20, 23, 25]. Referred to as rotating mapping codes, several methods have used such codes to eliminate homopolymers at the cost of a decrease in information-density. For example, in the method reported by Goldman et al. [15] a sequence of trits (base-3 values) is converted to a sequence of bases using the following mapping algorithm. The first trit in the sequence is mapped to a base according to {0,1,2} -> {C,G,T}, respectively. After the first base, the prior base is used to choose one of four direct mapping codes (“mapping scheme” in Fig. 2). The next trit is mapped to a base using this direct code. This process is repeated until the entire sequence has been encoded. This rotating mapping code causes a 25% drop in information density. However, other rotating codes have been designed that have higher information density of 1.75 bits/nt [23]. A novel rotating mapping code which utilizes a chained hash function that enables robust error correction was reported by Press et al. [25] and is discussed in greater detail in section "Error correction" below. The ability to choose the coding rate for this method enables one to increase information density at the cost of decreased error correction if necessary.

### Handling errors

DNA is a noisy communications channel in the sense that errors occur relatively frequently in this medium. Methods for storing digital data in DNA employ a variety of strategies to mitigate or correct these errors. The following notable strategies were found in the literature. Here, strategies for handling errors were generally divided into two categories: Error correction (i.e., strategies for identifying and correcting errors after they have occurred) or error mitigation (i.e., strategies for minimizing the occurance of errors). Most known methods simultaneously utilize both mitigation and correction strategies.

#### Expected errors

Errors encountered while storing information in DNA typically manifest as sequence dropout (an entirely missing sequence), single nucleotide variant (SNV) (substitution of a single base), indels (insertion or deletion of a single base), or truncations (removal of several bases from one end of a sequence). The following mechanisms are known to cause such errors: imperfect synthesis, degradation, imperfect polymerase chain reaction amplification, or imperfect sequencing. Error rates have been noted to depend on factors such as the exact synthesis, storage, and sequencing methods utilized. Overall, DNA as a storage medium exhibits much higher error rates than conventional storage medium (Table 1, right column). For example, Church et al [7] reported error rates of 1 bit per 0.7 megabytes (MBs), which is much higher than error rates of 1 bit per 10–1000 terabytes (TBs) exhibited by electronic memories. However, typical error rates may be much higher than this; Bornholt et al. [18] reported an error rate of approximately 1% and Organick et al. [20] reported error rates of up to 10%.

Certain structural patterns are known to exhibit higher synthesis and sequencing errors. Schwartz et al. [26] noted that base-sequences with more than 60% GC content exhibit higher dropout rates. Yazdi et al. [27] reported that base-sequences that maintaining  50% GC content ratio reduces synthesis/sequencing errors. Several studies have reported errors associated with homopolymer runs (such as AAAAA or TTTTTT). Specifically, Ross et al. [28] noted that homopolymer runs of more than 4 bases correlate with additional indel errors. Ananda et al. [29] noted PCR errors which rapidly increases with homopolymers greater than 4 bases. Both Poon et al. [30] and Xu et al. [31] noted that homopolymers of six or more bases exhibit high enough thermal stability to make sequencing difficult [30, 31].Chen et al. [32] discovered a relationship between spatial location on synthesis chips and synthesis bias, and also determined that PCR amplification can lead to stochastic variation, resulting in up to 2% data loss per amplification. Church et al. [7] noted their errors generally occurred within homopolymer runs near the end of sequences.Blawat et al. [17] observed that long sequences of repeated digital values in their data lead to periodically repeating 10-base sub-sequences and caused high dropout rates for the associated base-sequences.

#### Error correction

**Table 2:** Comparison of different error correction algorithms

	Repetition code	Hamming code	RS	BCH	LDPC
Error correction	Multiple bits	Single bit	Multiple bits	Multiple bits	Multiple bits
Error detection	Multiple bits	Two bits	Multiple bits	Multiple bits	Multiple bits
Error types	Scatter + burst	Scatter	Burst	Scatter	Scatter
Soft bit decoding	Yes	No	No	No	Yes

This table was adapted from the work of Regulapati [55]

Many known methods for storing digital data in DNA incorporate well-known algorithms for error detection or correction. The following notable strategies for detecting or correcting errors were found in the literature. Table 2 compares the most popular error correction algorithms used to store data in DNA, comparing both the types of errors they can detect/correct and how many bit errors they can detect/correct. This table utilizes Regulapati’s terminology to describe these errors, with indels corresponding roughly to burst errors and substitutions corresponding roughly to scatter errors.

Several known methods utilize repetition codes for error detection and correction [15, 18]. In repetition codes, a block of data is repeated multiple times. If during decoding, a data block is found to differ from the other copies of the same block, majority voting is used to determine the correct data block. Repetition codes are one of the most basic (and common) error correction strategies, and are naturally present in many known storage methods due to the fact that DNA synthesis techniques typically produce multiple copies of each DNA molecule. While repetition codes are simple, they are inefficient since only a fraction of the data blocks represent unique data. This leads to a decrease in information density. The method reported by Goldman et al. [15] utilized a repetition code where each data-segment was repeated 4 times, resulting in a 75% decrease in information density. The method reported by Bornholt et al. [18] utilizes a repetition code involving an exclusive-or operation where each segment is repeated 1.5 times, and the decrease in information density is approximately 33%.

Some known methods addressed errors using a Hamming code [24]. A hamming code is a member of the linear block code family, which was developed in 1950 by Richard W. Hamming [33]. Hamming codes can detect at most two bits of error in the data block and can fix one bit of error in the data block. Because of these limitations, hamming codes have rarely been used in DNA based storage systems. Takahashi et al. [24] used a (31, 26) hamming code for DNA data storage. However, errors like deletion and read truncation can make data retrieval difficult. Out of 25,592 reads reported by Takahashi, 16 were perfect reads and eight were corrupted but correctable.

Several known methods address errors using Reed Solomon (RS) codes [16, 17, 20–22, 34]. RS codes are a special class of BCH codes [35, 36]. These are widely used in error correction algorithms and are capable of correcting both burst errors and erasures. They have been widely applied for data storage or digital communication applications such as CD, DVD, QR codes, and mobile/satellite communications [37]. RS codes are more effective for indel errors rather than SNV errors. If the message size is n bytes, RS codes add redundant data of size k bytes at the end of the message. RS codes can detect k bytes of errors at arbitrary locations and can correct up to $\lfloor \frac{k}{2} \rfloor$ bytes of errors at arbitrary locations. One of the advantages of RS codes over the other error correction algorithms is the length of redundant code block *k* can be changed depending on the usage or by analyzing prior error patterns.

Methods which utilize RS codes include both the method reported by Erlich and Zielinski [21] and the method reported by Organick et al. [20]. In the method reported by Erlich and Zielinski [21], an RS code was applied within each sequence to verify the integrity of the sequence. This enabled this method to quickly discard corrupted sequences, which helped simplify decoding. In the method reported by Organick et al. [20], a RS code was applied to blocks of data such that new sequences just for error correction were introduced. These redundant sequences were observed to effectively handle sequence dropout and other high-level errors. However, it has been observed that RS codes work best for small data sizes [21, 38], and this approach necessitated relatively large data sizes.

Several known methods address errors using Low Density Parity Check (LDPC) codes. [39–41] LDPC [42] codes are a class of linear error correction codes developed by Robert G. Gallagher in 1962. Such codes have gained popularity because of their ability to provide the probability of a bit being 0 or 1, referred to as soft-decoding, which is sometimes advantageous over hard-bit decoding (where only a value of 0 or 1 is provided).

Known storage methods which utilize LDPC codes include the storage method reported by Shubham et al. [40]. This method utilizes three different error correction algorithms. At the first level, LDPC codes were used as the outer level error correction. The block size of each individual LDPC code was 32 kilobytes. In order to handle indel errors, a synchronization marker was inserted (the base-sequence “AGT”) in the middle of the data block. During the decoding process, if a sequence does not have a correct length the synchronization marker was used to recover the sequence. For example, if the synchronization marker is shifted left by 1 base, only the right part of the marker is considered valid. The authors observed the synchronization marker improved the reading performance by 10% and reduced information density by 2-3%.

The method reported by Press et al. [25] incorporates error correction into the mapping code and algorithm. This algorithm, referred to as the Hash Encoded Decoded by Greedy Exhaustive Search (HEDGES) algorithm, leverages the constraints of a hash algorithm-based rotating code to provide for robust error correction. In its simplest form a half rate code is used and the algorithm directly encodes each bit $b_i \in \{0,1\}$ by emitting a character $C_i \in \{A, C, G, T\}$ corresponding to $C_i = (k_i + b_i)$ modulo 4, where $K_i$ is the value of a hash function on some set of previously encoded bits. This reduces the information storage capacity by 50%, since at each step only two possible bases are available for encoding information. However, higher code-rates using the HEDGES algorithm are possible. One advantage of this approach is that it creates a highly dependent chain of encoded information that enables robust error correction. During data recovery, insertions, deletions, and mutations can be detected as violations of the chained hash values, and an attempt to correct them can be made via a minimum cost edit distance search, implemented with an A* style search algorithm. A final RS error code is utilized to check the output of the A* search, and to correct any remaining errors. The ability to choose the coding rate for this method enables one to increase error correction at the cost of information density if necessary.

Zhang et al. [43] developed a path-based error correction method that uses a saturated reverse search to guess the type of error and test three adjustment types (substitution, insertion, deletion) at each position in a local range. This method can be computationally inefficient due to the potential exponential growth of candidates. Also, this method is limited to correcting a single error, and multiple errors can only be fixed if they are isolated enough.

#### Error mitigation

Most known methods for DNA data storage avoided errors proactively using error mitigation strategies. The following paragraphs introduce several notable strategies which were found in the literature.

Several known methods utilized freedom afforded by the mapping algorithm to avoid extreme GC content, homopolymers, or long repeating sequences [7, 15, 16, 18, 20, 23].

The method reported by Organick *el al.* [20] additionally used rationally designed 20-base sequences as file addresses. The process used to generate these sequences avoided high GC contents, some secondary structures, and maintained a minimum hamming distance of six. These authors speculated that the current method could be used to generate a maximum library size of 14,000 20-mers. However, recently developed algorithms for generating large sets of orthogonal and experimentally viable primer sequences may be able to increase this maximum library size [44–49].

The methods reported by Erlich and Zielinski [21] and Anavy et al. [22] both take advantage of a fountain code to mitigate error-prone sequences. Since the fountain code enables them to randomly generate an arbitrary number of different encoding options, sequences that do not maintain a GC content ratio of 45-55% or that have long homopolymer runs are simply discarded. Zhang et al. [43] utilized a graph-based approach to reduce the occurrence of sequences that violate biological constraints. To achieve this, they proposed an algorithm called SPIDER-WEB, which initializes by screening out vertices that correspond to sequence violating the constraints. The algorithm then recursively trims the vertex set to improve its efficiency.

Several methods utilize either file compression or exclusive-or operations to eliminate structure in the data to store. For example, the method reported by Organick *el al.* [20] randomizes the input data by exclusive-or (XOR) with a pseudo-random sequence. As another example, the method reported by Takahashi et al. [24] appends the last 12 bits of the secure hash algorithm - 256 (SHA-256) hash of the original message to the original data, which is then XORed with a one time pad to increase the entropy of the data. This XOR operation reduces the possibility of repetition and hence long homopolymer runs.

## Performance considerations

In the following section, key performance considerations are identified and the advantages of different storage methods are discussed.

### Information density

For some applications, the highest possible information density is desired. Methods which store information in sequences of A/T/C/G have a theoretical maximum information density of 2 bits/base (or equivalently 2 bits/nucleotide) [7]. Several processes can decrease this information density, including: mapping algorithms, the addition of bases for addressing, or the addition of bases for error correction.

Of the in-vitro validated methods, the method reported by Erlich and Zielinski [21] exhibits the highest information density at 1.19 bits/base. (Calculation of this value included the primer sequences necessary for experimental validation, making this value lower than the 1.57 bits/base reported elsewhere.) The relatively high information density of this method can be explained by the coupling of several efficient components (i.e., the direct mapping code, the RS error correction code, and the fountain code with relatively low redundancy).

An alternative take on information density was proposed by Anavy et al. [22]. The associated method utilizes composite DNA letters to yield more bits per synthesis cycle than achievable using traditional A/T/C/G encodings. These authors reported in-silico densities as high as 6.4 bits per cycle, and in-vitro densities as high as 4.29 bits per cycle. However, the use of composite letters to store information has two key limitations. For one, effective use of composite letters depends on balancing the size of the composite alphabet with the tolerances of the sequencing and synthesis technologies. This presents an experimental limit on the composite alphabet size, and thus the logical density of the DNA, which will need to be addressed by improved sequencing and synthesis technologies. Additionally, the use of composite letters necessitates decreased physical data densities, which is evidenced by the tenfold decrease in physical density noted by the authors.

### Storage capacity

All known coding schemes have inherent limitations which restrict the maximum storage capacity. For most of these methods, this limitation arises from the finite number of addresses available to address data blocks. For such methods, the theoretical capacity is given by $B\cdot 2^{n}$, where *n* is the number of bits in the address system and B is the number of bytes stored per address. For instance, if we employ a storage system that uses 8 bits for addressing, with each address referring to a data block of size 32 bytes, then the overall capacity of the system would be given by $32 * 2^{n}$ bytes.

The storage method with the highest demonstrated capacity was reported by Organick et al. [20] and was used to store 200 megabytes of data. This method also has the highest theoretical capacity, and may be able to store multiple terabytes of data. Further, it is possible that recently developed algorithms for generating larger sets of address base-sequences may help further boost this capacity [44–49].

### Random access

Random access provides the ability to read specific information from storage. For applications where a large number of independent files are stored, it may be desirable to recover only select files in a given read cycle. However, several of the known methods require one to recover all the data before any can be accessed. Alternatively, some schemes are well suited for efficiently accessing just part of the stored data, potentially reducing sequencing time and costs.

One method with relatively high random-access is the method reported by Bornholt et al. [18]. This method uses a key-value scheme to allow random access to the data. This technique uses PCR to amplify only the desired data. For each DNA oligo, the key corresponds to the lower part of the address, and the value corresponds to the stored data. The key points to a small set of DNA strands that share that initial part of the address. Then, the primers are designed to match with the key, so by adding the desired primers, the strands addressed by them will be amplified, and after sampling, the majority of the sample corresponds to the target data.

The method reported by Organick et al. [20] allows an even higher degree of random access by including two addresses per DNA strand: One named File-ID (which uses a 20-base sequence as an address and groups the strands which belong to the same file), and one strand-specific address (which indicates data block and column index). Random access is achieved by using PCR to amplify only strands with primers that match the File-ID.

In their study, Banal et al. [50] introduced a novel method for random access that does not require PCR amplification. The system encodes information in DNA sequences, which are encapsulated in silica particles. Each file contains a file sequence and addressing barcodes used to identify the file via hybridization. Unique single-stranded DNA barcodes label the files and enable Boolean-logic-based selection on the entire data pool via simple hybridization. The system uses physical sorting to retrieve specific files or arbitrary subsets of files, without requiring amplification. To select target subsets of the complete data pool, fluorescence-activated sorting (FAS) is employed by annealing fluorescent oligonucleotide probes that are complementary to the barcodes used to address the database.

### Rewrite ability

**Fig. 9:**
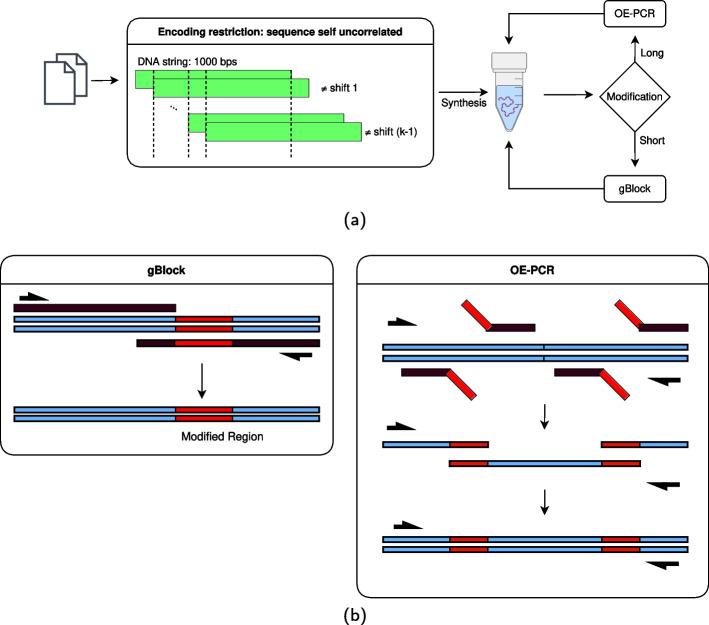
Diagram of the storage method reported by Yazdi et al. **a.** The data was encoded in blocks of 1000 bps, in which the beginning and ending 20 bps correspond to address strings, and the remaining 960 bps are composed of 12 sub-blocks of 80 bps that encode the digital data on six code words. The encoding is done such that the sequence is uncorrelated with itself, i.e. no shifts will overlap the sequence with itself **b.** DNA rewriting procedures. The gBlock method for short rewritings and OE-PCR for longer blocks

Most known methods for storing digital data in DNA base-sequences use a write-once process and can not be edited once written. However, the method proposed by Yazdi et al. [27] reported a novel DNA-based system capable of rewriting data at arbitrary locations (Fig. 9). To accomplish this, data was logically organized into blocks and indexed by address strings. The address strings are encoded to maximize Hamming distance. In this method, rewriting is accomplished using the gBlock or Overlap Extension Polymerase Chain Reaction (OE-PCR) method, which are used for long or short strings respectively as shown in Fig. 9b. Lin et al. [51] showed a set of file operations that can be performed within the DNA storage system, including locking, unlocking, renaming, and deleting. The renaming process involves mixing the original file strand with a 40 nt single-stranded DNA oligo that binds to the original file’s address, resulting in a new overhang that corresponds to the new address of the renamed file. This process ensures that only the accessing oligo with the overhang complementary to the new address can effectively separate the file strands, while also blocking any other oligos that are not designed for the renamed file. Deletion uses a 20 nt oligo complementary to the file’s original address to block the overhang of the file strand or extract it from the database. This ensures no leftover strands are spuriously accessed in the future.

### Cost

Although storing data in DNA has several advantages over current storage media, cost remains one of the most prominent disadvantages. One estimate indicates the current cost to store data using DNA is approximately $800 million per TB [52], while tape costs is approximately $16 per TB [53]. However, DNA synthesis and sequencing costs are dropping at an exponential rate of 5- and 12-fold per year respectively [7, 54]. The cost of storing data into DNA directly depends on the efficiency of the encoding/decoding algorithm. In 2013 Goldman et al. [15] required USD 12,400 to encode 1 megabyte while their encoding efficiency was 0.88 bits/base. However, if they were able to achieve an encoding efficiency of 0.94 bits/base their cost would have been reduced to 7440 USD/MB. In 2017 Erlich and Zielinski [21] encoded information at the cost of  3500 USD/megabyte, which is almost one-fourth the cost of Goldman et al. [15]. In 2019, Anavy et al. [22] used composite DNA in the synthesis cycle, which increased the logical density of the DNA and further reduced the cost by up to 52% to an (estimated) cost of  1,700 USD/MB. Although the cost of writing data to DNA remains much higher than established methods such as tape, the maintenance cost of DNA is significantly lower once written.

## Conclusion

**Table 3:** A comparison of selected methods for storing information in DNA

Method	File size (MB)	Error handling	Information density (bits/base)	Physical density (PB/g)	Key contribution
Church et al. [7]	0.65	Repetition	0.60	1.28	Increased data capacity
Goldman et al. [15]	0.75	Repetition	0.33	2.25	Introduced data redundancy
Grass et al. [16]	0.08	RS	1.14	25	Utilized RS code, reduced data redundancy
Yazdi et al. [27]	0.017	Yes	–	$4.9 \times 10^{5}$	Increased random access, introduced rewrite ability
Blawat et al. [17]	22	RS	0.92	–	Error free retrieval of larger scale data
Bornholt et al. [18]	0.15	Repetition	0.88	–	Reduced redundancy, increased random access
Erlich and Zielinski [21]	2.14	Fountain & RS	1.19	214	First fountain code, increased data density
Organick et al. [20]	200.2	RS	1.1	–	Increased data capacity
Anavy et al. [22]	21.4	Fountain & RS	–	20–30	Introduced composite DNA letters

This table was adapted from the work of Erlich and Zielinski [21]

Numerous in-vitro validated methods for storing digital data in DNA have been reported. These methods address a several recurring themes (i.e., mapping to base-sequence and handling of errors) using a variety of strategies. We find the relative advantages and disadvantages of the methods to be highly application specific. For example, some methods have relatively high information densities [16, 20, 21], some methods provide a relatively high degree of random-access [18, 20], and some provide relatively high physical densities [21, 27]. Table 3 summarizes key criteria for select methods.

Unfortunately, none of the discussed methods are suitable for storing the magnitude of data necessary to address the potential information storage crisis. However, several key advancements may help make DNA data storage more cost effective. Foremost, the cost of DNA synthesis must be reduced or coding schemes that are more efficient per synthesis cycle must be developed (similar in principle to the work of Anavy et al. [22]). Approaches for addressing the later objective include the development of more effective error mitigation methods or more efficient error correction methods. Promising methods for improving both error mitigation and error-correction have been proposed, but it is critical that these methods be validated in-vitro so that their performance can be fairly assessed. For error-mitigation, this includes methods which focus on decreasing synthesis errors, improving avoidance of problematic sequences, and decreasing sequencing errors. For error-correction, this includes methods which focus on increased efficiency such that high information densities can be maintained.

## Data Availability

Not applicable.

## Abbreviations

A: Adenine
BCH: Bose-chaudhuri-hocquenghem
bp: Base-pair
C: Cytosine
CRC: Cyclic redundancy check
DNA: Deoxyribonucleic acid
EB: Exabyte
ECC: Error correction code
G: Guanine
GF: Galois field
LDPC: Low density parity check
LFSR: Linear-feedback shift register
MB: Megabyte
MSA: Multiple sequence alignment
nt: Nucleotide
OE-PCR: Overlap extension polymerase chain reaction
oligo: Oligonucleotide
PB: Petabyte
PCR: Polymerase chain reaction
PRNG: Pseudorandom number generator
RS: Reed-solomon
SHA-256: Secure hash algorithm - 256
T: Thymine
TB: Terabyte
XOR: Exclusive-OR
ZB: Zettabyte
